# Multi-Event Dynamics Modeling and Bidirectional Radial Response Analysis of Localized Groove Defects on the Outer Ring of Rolling Element Bearings

**DOI:** 10.3390/ma19194046

**Published:** 2026-09-22

**Authors:** Kai Chai, Shuyong Liu, Taoming Yang

**Affiliations:** College of Naval Architecture and Ocean Engineering, Naval University of Engineering, Wuhan 430033, China; hjcckll@sina.com (S.L.); hjczsj@163.com (T.Y.)

**Keywords:** rolling element bearing, outer-ring groove defect, geometric contact, multi-event dynamics, bidirectional radial response, experimental trend comparison

## Abstract

**Highlights:**

**What are the main findings?**

- 3D groove contact reproduces load release, separation, edge impact, and contact recovery.
- Speed governs defect-passing frequency; groove width and radial load mainly change amplitude.
- Circumferential position shifts the dominant radial response between the Y and Z directions.

**What are the implications of the main findings?**

- Frequency, amplitude, and directional information should be combined for defect assessment.
- The model supports mechanism and trend interpretation rather than absolute amplitude calibration.

**Abstract:**

Localized bearing defects are often modeled as imposed displacements or instantaneous impacts, limiting continuous description of contact-state evolution. This study develops a multi-event ADAMS model for an axial-through outer-ring groove in an SKF 6208 bearing. The model retains rolling-element translational inertia, compliant Hertz/IMPACT contacts, and geometry-driven contact transitions. Relative angular position, local clearance, and contact deformation label the event states, while the continuous equations of motion determine the forces and trajectories. A directional sensitivity index uses dominant peaks around the defect fundamental and second harmonic. The model reproduces load release, short-term separation, trailing-edge impact, and contact recovery. At 1800 r/min, reconstructed experimental pulse-interval errors for 0.5, 1.0, and 1.5 mm grooves are 3.13%, 1.84%, and 0.86%, respectively. Speed governs passing frequency, whereas defect width and radial load mainly affect the simulated spectral-peak amplitude; circumferential position redistributes the response between the Y and Z directions. The expected event sequence and frequency/directional trends were confirmed. Absolute housing-vibration amplitudes and the magnitude of physical contact disruption were not calibrated: the component bodies and support path are rigid, the contact parameters were not identified from tests, and simulation displacement is compared with housing acceleration under different loads. The experimental comparison is therefore limited to pulse-period consistency and phenomenological parameter trends.

## 1. Introduction

Rolling element bearings support rotating components, transmit loads, and constrain motion. Localized defects generate impacts and modulated vibration through periodic changes in contact state. Pulse-sequence models have low computational cost but prescribe the excitation; analytical bearing models resolve a limited number of generalized coordinates; finite-element models resolve local deformation at substantially higher cost. The present multibody model occupies an intermediate level: 12 component bodies, approximately 63 degrees of freedom, and 27 candidate ball-ring/cage contacts are evaluated at each integration step, so the contact workload scales linearly with the number of rolling elements and no spatial finite-element mesh is required [1,2]. This structure links three-dimensional defect geometry, contact-state evolution, and observable response trends while retaining tractable parameter sweeps.

Bearing dynamics research has progressed from rolling-element motion and sliding analyses to multi-degree-of-freedom models containing inner and outer rings, rolling elements, and cages. Jones established the framework for ball motion and sliding friction [3], Harris and Mindel studied rolling-element/raceway dynamics [4], Gupta developed a three-dimensional ball-bearing analysis [5], and Sunnersjo revealed varying-compliance vibration caused by a finite number of rolling elements [6]. These studies established the foundations for load distribution, contact deformation, and rolling-element motion. However, when a localized defect is represented only by an imposed displacement or preset pulse, load release, loss of contact, edge collision, and contact recovery remain difficult to describe continuously within one model.

Recent localized-defect models have advanced experimental comparison, three-dimensional geometry, and instantaneous contact description. Mattar et al. established nonlinear inner- and outer-ring defect models with experiments [7]; Gao et al. developed a three-dimensional cubic-defect model for an angular-contact ball bearing [8]; Mufazzal et al. analyzed localized outer-ring defects and extended defects [9,10]; Cao et al. formulated instantaneous contact from component positions [11]; Li et al. considered irregular morphology [12]; Guo et al. analyzed time-varying stiffness [13]; Giraudo et al. developed a multibody model with experimental comparison [14]; and Wen et al. examined the effects of defect size and location on impact detectability [15]. Xu and Chen included a random defect base, centrifugal force, and gyroscopic moment [16], whereas Wang et al. used finite-size progressive contact and LuGre friction to describe entry, main-contact, and exit phases [17]. Tribological studies further show that rolling-contact response depends on lubricant chemistry, film formation, and advanced particulate additives: hydrocarbon formulations can change rolling-contact surface response [18], and the shape and concentration of Al_2_O_3_ or TiO_2_ nano-additives alter friction, wear, and load-carrying behavior [19,20]. These findings motivate explicit limits on any dry/equivalent contact model used to interpret vibration amplitude.

Mathematically, the ADAMS calculation is a continuous-time constrained multibody system. The labels contact, release, separation, edge impact, and recovery are diagnostic event classes defined by zero crossings of relative angle, clearance, and penetration; they do not form an independent discrete map and do not reset displacement or velocity. Numerical entry into the groove is regularized by the finite three-dimensional edge geometry, the penetration-dependent Hertz/IMPACT law, and the smoothed damping transition. Consequently, no discontinuous displacement excitation is imposed when a ball crosses the leading edge.

The study addresses the absence of a unified comparison of complete defect passage, bidirectional radial response, and experimentally observable periodicity for a fixed outer-ring axial-through groove. The following three expectations are tested: groove passage should produce the ordered sequence load release-separation-trailing-edge impact-recovery; speed should govern the defect-passing frequency while width and radial load mainly govern response magnitude; and circumferential position should redistribute response between orthogonal radial directions. The contribution is a three-dimensional geometry-to-event-to-direction framework: contact timing and magnitude are solved from the physical groove rather than a reconstructed displacement excitation, and a directional index connects the resolved events to Y/Z response redistribution. This focused contribution complements, rather than replaces, progressive analytical contact models such as [21,22], and is relevant to Materials because it makes the limits imposed by contact compliance, surface condition, and transfer-path material behavior explicit.

## 2. Materials and Methods

### 2.1. Motion Relationships and Basic Assumptions

The study considers an SKF 6208 deep-groove ball bearing (SKF, Gothenburg, Sweden) with the outer ring stationary relative to the spindle and the inner ring rotating with the spindle. The *X*-axis is axial, the *Y*-axis is the radial-load direction, and the *Z*-axis is the orthogonal radial direction. The outer ring is constrained against rotation; its reference point retains small Y/Z motion for comparing the internal distribution of the simulated response. These displacements are not equivalent to acceleration amplitudes measured on the bearing housing. The inner ring, outer ring, balls, and cage are modeled as rigid component bodies, whereas local normal compliance is represented by the Hertz/IMPACT contact law. Flexible housing-spindle deformation, material plasticity, and a resolved lubricant film are outside the model. The defect is an axial-through groove of width *w_d_* and depth *h_d_*, with *h_d_* sufficiently large to avoid ball-bottom contact. To expose dynamic slip and axial deformation in the kinematic interpretation, the cage angular velocity and the position of ball j are written in generalized form as

(1) $$\omega_{c}(t)=\frac{\omega_{i}}{2}\left[1-\frac{d_{b}}{D_{m}}\cos\;\alpha_{eff}(t)\right]\left[1-s_{c}(t)\right]$$

(2) $$\theta_{j}(t)=\theta_{0}+\int_{0}^{t}\omega_{c}(t)d\tau +\frac{2\pi (j-1)}{N_{b}},\;\;j=1,2,\cdots ,N_{b}$$

In Equations (1) and (2), *ω_i_* is the inner-ring angular velocity, *d_b_* is the ball diameter, *D_m_* is the pitch diameter, *N_b_* is the number of balls, *θ_0_* is the initial phase, *s_c_*(t) is the dynamic orbital slip factor, and *α_eff_*(t) is the effective contact angle that reflects the axial contact-deformation component. The integral in Equation (2) allows both quantities to vary with time. These equations provide a kinematic interpretation only; the event criterion uses the component coordinates solved by MSC ADAMS2020 rather than prescribing *θ_j_*(t). Ball mass and translational inertia are retained, so centrifugal and collision forces arise in the Newton–Euler solution. A centrifugal-force term is therefore not added to Equation (1), which is a kinematic relation rather than a force balance. For the nominal BPFO values in Section 3.4, *s_c_* = 0 and *α* = 0 are used only as an analytical reference.

### 2.2. Defect-Region State Switching and Contact Force

Let *θ_d_* denote the central angular position of the outer-ring groove, *w_d_* the circumferential groove width, and *R_o_* the outer-raceway radius. The defect half-angle *β_d_* and the angular position *φ_j_* of ball j relative to the groove are

(3) $$\beta_{d}=\frac{\omega_{d}}{2R_{0}},\;\phi_{j}(t)=wrap_{\left[-\pi ,\pi \right]}\left[\theta_{j}(t)-\theta_{d}\right]$$

The operator wrap [-π,π) maps an arbitrary angle to the principal interval [-pi, pi), so the relative angle remains continuous across the 0/2π coordinate boundary. The same symbols *θ_d_*, *w_d_*, *h_d_*, *β_d_*, and *φ_j_* are used in Figure 1 and Table 1; no additional geometric parameters are introduced in the figure.

Using the Heaviside function to represent the local clearance increment caused by the groove geometry, the equivalent contact deformation between the rolling element and the outer raceway is expressed as

(4) $$\delta_{j}\left(t\right)=\delta_{j0}\left(t\right)-h_{d}H(\beta_{d}-\left|\phi_{j}\left(t\right)\right|)$$

In the formula, $\delta_{j0}$ is the contact deformation without defect, and $h_{d}$ is the defect depth. When the contact deformation is positive, normal contact force is generated; the contact force is zero during separation:

(5) $$F_{n,j}=\left\{\begin{array}{cc} K\delta_{j}^{e}+C{\dot{\delta }}_{j}S\left(\delta_{j}\right), & \delta_{j}>0\\ 0, & \delta_{j}\leq 0 \end{array}\right.$$

Here K, e, and C are the equivalent contact stiffness, force exponent, and damping coefficient, respectively, and S(.) is the smoothed penetration-dependent transition used to ramp damping from zero to its full value. K and e define local elastic contact even though the component bodies are rigid; for ball-raceway contact, e = 1.5 is the Hertzian exponent, whereas the ball-cage value e = 2.2 is an equivalent model input. C and *d_max_* regularize damping near first contact and avoid an impulsive force jump. Equations (3)–(5) define local clearance and diagnostic event states; they are not external switching functions imposed on ADAMS. At each integration step, the physical three-dimensional groove, local contact normal, and IMPACT law in Section 2.3 determine force. The leading edge releases load, the groove permits momentary separation, the trailing edge changes the local normal and produces compression, and normal contact then recovers. Thus, the continuous mechanics generate the event sequence without a prescribed impact time or amplitude.

Within each integration step, the event state is evaluated from the solved relative angular position and contact penetration, and the ball-inner-raceway, ball-outer-raceway, and ball-cage contact forces are projected into the global coordinate system to assemble the Newton–Euler equations. Because ball mass and translational acceleration are retained, inertial and centrifugal effects participate directly in the edge-collision solution. The model therefore describes normal contact-load release-short-term separation-trailing-edge impact-contact recovery without prescribing impact timing or magnitude. Table 1 is a diagnostic classification of the continuous solution, not a discrete switching program that replaces geometric contact.

### 2.3. ADAMS Model Structure and Contact Parameters

The SKF 6208 geometry is listed in Table 2. The three-dimensional solid model imported into ADAMS contains the inner ring, outer ring, cage, and nine balls. The component bodies are rigid, but 27 compliant candidate contacts are evaluated as follows: nine ball-inner-raceway contacts, nine ball-outer-raceway contacts, and nine ball-cage contacts. The outer ring is constrained against rotation and rigid-body drift; radial load acts along Y, Z is the orthogonal radial direction, and the inner ring is speed driven. Y/Z displacements are extracted at the outer-ring reference point. The software constraint statistics give approximately 63 degrees of freedom, with no over-constraint or undefined component. The model geometry, boundary conditions, component bodies, and compliant contact-pair topology are illustrated in Figure 2.

ADAMS contact uses the IMPACT function, whose equivalent normal force form is

(6) $$F=Kg^{e}+C\dot{g}\mathrm{STEP}\left(g,0,0,d_{max},1\right),g>0$$

In Equation (6), g is contact penetration and *d_max_* is the penetration at which damping reaches C. Table 3 lists the deterministic inputs retained from the original ADAMS model. K is equivalent normal stiffness, e is the force exponent, C is the maximum damping coefficient, and *μ_s_* and *μ_d_* are the static and dynamic friction coefficients. The value *μ_s_* = 0.30 for ball-cage contact is a conservative equivalent guidance-contact input from the original model, not a measured lubricated coefficient. No separate lubricant film is defined between the cage and balls; lubrication, shear thinning, and temperature are therefore lumped into the effective friction and damping inputs. Because K, C, and the friction coefficients were not identified by independent contact tests, the present study uses them for mechanism and parameter-trend analysis and does not treat simulated displacement amplitude as an absolute cross-rig prediction.

## 3. Results

### 3.1. Numerical Consistency Under the Reference Condition

The reference condition is 1800 r/min spindle speed, 1500 N radial load, and 300 N axial load. The simulation duration is 1.0 s, and the output interval is 0.001 s, giving a 1000 Hz output sampling frequency and 500 Hz Nyquist frequency. Quantitative spectral statistics therefore use only the BPFO fundamental and second harmonic; higher-frequency collision details are not used quantitatively. After removal of startup data, the cage trajectory converges and ball motion is periodic. From the mean ADAMS orbital speed, the reference-condition orbital slip ratio is calculated as *s_c_* = |*ω_c,kin_*-mean(*ω_c_*)|/*ω_c,kin_* × 100% = 1.72%. This is an uncalibrated numerical diagnostic: ball/cage speed was not measured experimentally, and no parameter-dependent slip-ratio trend is used to support the conclusions. Raceway contact forces are of order hundreds of newtons, while cage contact forces are of order tens of newtons, consistent with primary load transmission through the raceways. These checks establish numerical consistency, not experimental validation.

### 3.2. Defect-Passing Process

A 5 mm axial-through groove is placed in the maximum load zone to make the complete event sequence visually separable in the reference trajectory. This width is an intentionally exaggerated parametric upper bound, not the IMPACT penetration *d_max_* and not the groove depth *h_d_*. The experimental grooves are 0.5–1.5 mm wide and 1.0 mm deep, whereas the numerical width sweep spans 0.5–5.0 mm. Figure 3 shows that load is released at the leading edge, contact is briefly lost inside the groove, the ball collides with the trailing edge, and normal contact recovers. The Y component is larger in the maximum load zone, while Z reflects the orthogonal component. With the 0.001 s output interval, the event order is resolved but high-frequency collision-waveform details are not quantified.

### 3.3. Experimental Trend Comparison

BVT-5 vibration data are used only to compare defect-passing periodicity and broad parameter trends. The test bearing is an SKF 6208 with 0.5, 1.0, and 1.5 mm axial-through outer-ring raceway grooves of 1.0 mm depth. Test loads are 300 N radial and 100 N axial, while the simulation reference loads are 1500 N radial and 300 N axial. The measured quantity is housing acceleration sampled at 65,536 Hz; the simulated quantity is outer-ring reference-point displacement output at 1000 Hz. Pulse sequences are reconstructed from the steady-state test signals and their mean adjacent-pulse interval is calculated. Because load, response quantity, transfer path, and bandwidth are unmatched, neither an absolute amplitude error nor inverse identification of ADAMS contact parameters is reported. Table 4 is restricted to pulse-period reconstruction at 1800 r/min.

At 1800 r/min, the nominal BPFO is 108 Hz and the theoretical pulse interval is 9.26 ms. The reconstructed experimental intervals give pulse-interval errors of 3.13%, 1.84%, and 0.86%. This metric compares reconstructed pulse period with the nominal theoretical period; it is neither a slip-ratio error nor an ADAMS amplitude-prediction error. Increasing groove width improves periodic-impact identifiability in the experimental signal. These observations support the frequency and trend interpretation only and do not constitute full validation under matched conditions.

### 3.4. Response Indicators and Characteristic Frequencies

Spectrum analysis is performed after removing the mean from the displacements in the two orthogonal radial directions Y and Z at the outer ring reference point. *X_q_*(*f*) represents the spectrum of displacement in direction *q*. Considering the 0.001 s output interval corresponding to a 500 Hz Nyquist frequency, this paper only sets search bands Ω*_k_* = [*kf_o_* − Δ*f*, *kf_o_* + Δ*f*] (*k* = 1, 2) near the outer ring defect fundamental frequency and double frequency, defining the defect-related spectral peaks within each harmonic band as

(7) $$A_{kq}=\max_{f\in \Omega_{k}}\left|X_{q}(f)\right|,\;\;\;\;\;k=1,2,\;q\in \left\{Y,Z\right\}$$

(8) $$A_{q}=\max\left|A_{1q},A_{2q}\right|$$

where *A_q_* denotes the dominant defect-related spectral peak at the fundamental and double frequencies in direction *q*. The directional sensitivity index is defined as

(9) $$I_{d}=\frac{A_{Y}-A_{Z}}{A_{Y}+A_{Z}}$$

where Id > 0 indicates that the response in the Y direction is dominant, Id < 0 indicates that the Z direction is dominant, and Id close to 0 indicates comparable amplitudes in both directions. Since AY and AZ use the same harmonic range and search bandwidth, the index mainly reflects the relative response distribution between the two orthogonal radial directions, rather than directly estimating defect size. The characteristic frequency of an outer ring localized defect is

(10) $$f_{o}=\frac{N_{b}}{2}\left(1-\frac{d_{b}}{D_{m}}\cos\;\alpha \right)f_{r}$$

Here *N_b_*, *d_b_*, *D_m_*, alpha, and f_r denote ball count, ball diameter, pitch diameter, contact angle, and rotational frequency. Table 5 uses the nominal geometric reference *N_b_* = 9, *d_b_* = 12 mm, *D_m_* = 60 mm, and *α* = 0, giving f_o = 3.6f_r. The generalized kinematics in Equations (1) and (2) retain *α_eff_*(t) and dynamic slip, but neither was experimentally identified for the present rig. A sensitivity illustration shows the scale of the contact-angle correction: at 1800 r/min, substituting *α* = 15 degrees into Equation (10) gives 108.92 Hz instead of 108.00 Hz, an increase of 0.85%; 15 degrees is an illustration, not an estimate of the actual operating angle. The uncalibrated axial-load-induced contact-angle variation remains a stated model boundary.

**Table 5:** Nominal theoretical outer-ring raceway defect frequencies at representative speeds (*α* = 0).

Spindle Speed/(r/min)	Rotational Frequency/Hz	Ball Pass Frequency of Outer Race (BPFO)/Hz
1200	20	72
1500	25	90
1800	30	108
2100	35	126

### 3.5. Effects of Defect Width and Circumferential Position

Under the reference condition, groove width is varied from 0.5 to 5.0 mm in 0.5 mm increments. The 0.5–1.5 mm range corresponds to the tested defects; 2.0–5.0 mm is a numerical extension used to reveal the loss-of-contact and trailing-edge impact trends. Figure 4c shows the defect-related dominant spectral peak rising from approximately 2.2 × 10^−3^ to 6.6 × 10^−3^ mm while its frequency remains near 108 Hz and low-order harmonics. The 5 mm endpoint is a width parameter only and is independent of contact penetration *d_max_* = 0.01 mm in Table 3.

With the defect width maintained at 5 mm, the defect center is circumferentially offset from 0° to 90° relative to the maximum load position, in 10° increments. Figure 4d shows that the overall amplitude in both directions decreases as the offset angle increases; the amplitude in the Y direction decays faster than in the Z direction, with the two directions having comparable amplitude around 40°, after which the Z direction dominates. The defect circumferential position does not change the characteristic frequency of 108 Hz but alters the local load-bearing magnitude and the load distribution in the two orthogonal radial directions.

### 3.6. Effects of Spindle Speed and Radial Load

To study the trend over a wide range of operating conditions, the spindle speed was increased from 300 r/min to 3000 r/min in 300 r/min increments. Figure 4a shows that the increase in speed shortens the defect passing interval, and the characteristic frequency increases linearly according to Equation (10). The defect-related dominant spectral peak amplitude generally increases, with a more significant rise in the high-speed range. At 3000 r/min, the outer ring defect fundamental frequency and double frequency are 180 Hz and 360 Hz respectively, both within the resolvable range of the data presented here. This trend is consistent with the effect of simultaneous increase in Defect Passing Frequency and the relative collision velocity of the trailing edge.

With the spindle speed maintained at 1800 r/min, axial load at 300 N, and defect width of 5 mm, the radial load is increased from 300 N to 3000 N in steps of 300 N. Figure 4b shows that the defect-related spectral peak amplitude increases monotonically with radial load, while the spectral line position remains near 108 Hz and its harmonics. The results indicate that radial load mainly modulates the response amplitude by increasing contact deformation and normal collision force at the trailing edge.

### 3.7. Parameter Influence Patterns

From Figure 4a–d and the prior parameter analysis, it is evident that the effect pathways of defect geometry parameters and operating conditions on the outer ring response differ significantly. Their influence on characteristic frequency, dominant defect-related spectral peak, and response direction is summarized in Table 6. Spindle speed is the primary factor altering the outer ring defect characteristic frequency; defect width and radial load mainly modulate the spectral peak amplitude by changing the loss of contact process, trailing edge impact, and contact load, without affecting the characteristic frequency. In addition to affecting the overall response amplitude, the defect circumferential position also alters the distribution of load in the two orthogonal radial directions, causing the directional sensitivity index to gradually approach and cross zero, with the dominant response shifting from the Y direction to the Z direction. Therefore, outer ring defect identification should jointly consider frequency, amplitude, and directional information and compensate for changes in speed and load.

## 4. Discussion

### 4.1. Multi-Event Formation Mechanism During Complete Defect Passage

Prior studies describe leading-edge entry, trailing-edge impact, load-zone dependence, instantaneous contact, extended defects, multi-event excitation, and spall-ball interaction [9,10,11,21,22]. Wang et al. [17] further resolve pre-entry, main-contact, and exit phases with finite-size progressive contact and nonlinear friction. The present model is narrower: it addresses a fixed outer-ring axial-through groove and uses solved geometry and contact penetration to align the continuous contact-force history with five interpretable event classes. Its concrete contribution is the linkage between those geometry-generated events and bidirectional Y/Z response redistribution, including the position-dependent change in dominant direction. It does not claim to replace progressive analytical contact laws or to provide a new absolute-amplitude calibration.

### 4.2. Feature Interpretation, Engineering Implications, and Limitations

The outer-ring defect response contains frequency, amplitude, and directional information. Nominal BPFO, ADAMS spectral lines, and reconstructed pulse intervals support defect-type and speed-tracking interpretation. Simulated spectral-peak amplitude is affected by width, speed, load, contact parameters, and transfer path, so it should not be used as an operating-condition-independent severity measure. The directional index is more sensitive to groove position. For monitoring, two orthogonal radial measurement directions and speed-synchronous/order analysis should be combined with load-conditioned amplitude baselines and the fundamental/second-harmonic bands.

A quantitative bridge to absolute housing acceleration requires matched operating conditions and an identified transfer path. In the frequency domain, a practical calibration relation is

$$\partial_{preq,q}(f)=-{\left(2\pi f\right)}^{2}H_{q}(f)x_{model}{,}_{q}(f)$$

where *x_model,q_* is the simulated outer-ring displacement and *H_q_* is the housing-spindle transfer function identified from impact or operational response tests in direction q. The contact/lubrication parameter vector can then be identified by minimizing a weighted two-direction error over selected frequency bands and operating points. Reporting normalized root-mean-square error, amplitude/phase error at BPFO harmonics, and coherence on held-out speed/load conditions would distinguish contact-parameter error from transfer-path error. The current unmatched data do not support this calibration, so no amplitude-error percentage is reported.

Lubrication can materially reduce edge-impact amplitude. A lubricant film introduces squeeze-film damping and speed-, load-, temperature-, and viscosity-dependent shear; surface chemistry and advanced nano-additives can also alter friction, film formation, and wear [18,19,20]. The present C, *d_max_*, *μ_s_*, and *μ_d_* values only lump these effects into equivalent dry/contact inputs. Consequently, the model may overestimate the sharpness and transmitted magnitude of groove-edge impacts under full-film or mixed-lubrication conditions, although the geometry-controlled event order and nominal passing frequency should remain. A coupled elastohydrodynamic-contact model and lubricant-specific parameter identification are required before amplitude calibration.

The model retains important limitations. Component bodies are rigid and the flexible housing-spindle transfer path is excluded; only local contact is compliant. Lubrication, temperature, random surface morphology, plasticity, and irregular groove geometry are not resolved. Dynamic slip and the axial-load-induced effective contact angle are not experimentally identified, and the 1.72% slip ratio is only a reference-condition numerical diagnostic. The parameter study is single-factor, the 5 mm groove is an exaggerated numerical endpoint, and the 0.001 s output interval does not support quantitative high-frequency collision analysis. Finally, the tests use different loads, acceleration rather than displacement, and a different transfer path. The conclusions are therefore restricted to event mechanism, nominal frequency consistency, and parameter trends; matched bidirectional tests, flexible-body coupling, slip measurements, and lubricant-specific calibration remain necessary.

## 5. Conclusions

(1) The results confirmed the first expectation: geometry-resolved ADAMS contact reproduced the ordered sequence of load release, short separation, trailing-edge impact, and contact recovery, while the principal spectral lines remained consistent with the nominal outer-ring raceway defect frequency. The reconstructed experimental pulse-interval error at 1800 r/min was 0.86–3.13%. This is pulse-period consistency, not displacement-amplitude prediction accuracy.
(2) The width study confirmed the second expectation within the modeled range: increasing groove width from 0.5 to 5.0 mm increased the dominant simulated BPFO-band peak from approximately 2.2 × 10^−3^ to 6.6 × 10^−3^ mm without materially changing its frequency. The 5 mm case is an exaggerated numerical endpoint used to resolve the event sequence.
(3) The position study confirmed the third expectation: moving the groove away from the maximum load zone weakened both radial responses, with Y decreasing faster than Z. Near 40 degrees the directional index approached zero, after which the dominant simulated response changed from Y to Z.
(4) The expected speed and load trends were also confirmed: speed changed both nominal characteristic frequency and simulated response magnitude, whereas radial load mainly changed magnitude. No opposite trend was observed within the reported parameter ranges. The following two capabilities were not confirmed: absolute housing-acceleration prediction and experimental validation of slip. These require matched loads and measurement quantities, an identified flexible transfer function, lubricant-specific contact parameters, and direct ball/cage kinematic measurements.

## Figures and Tables

**Figure 1:**
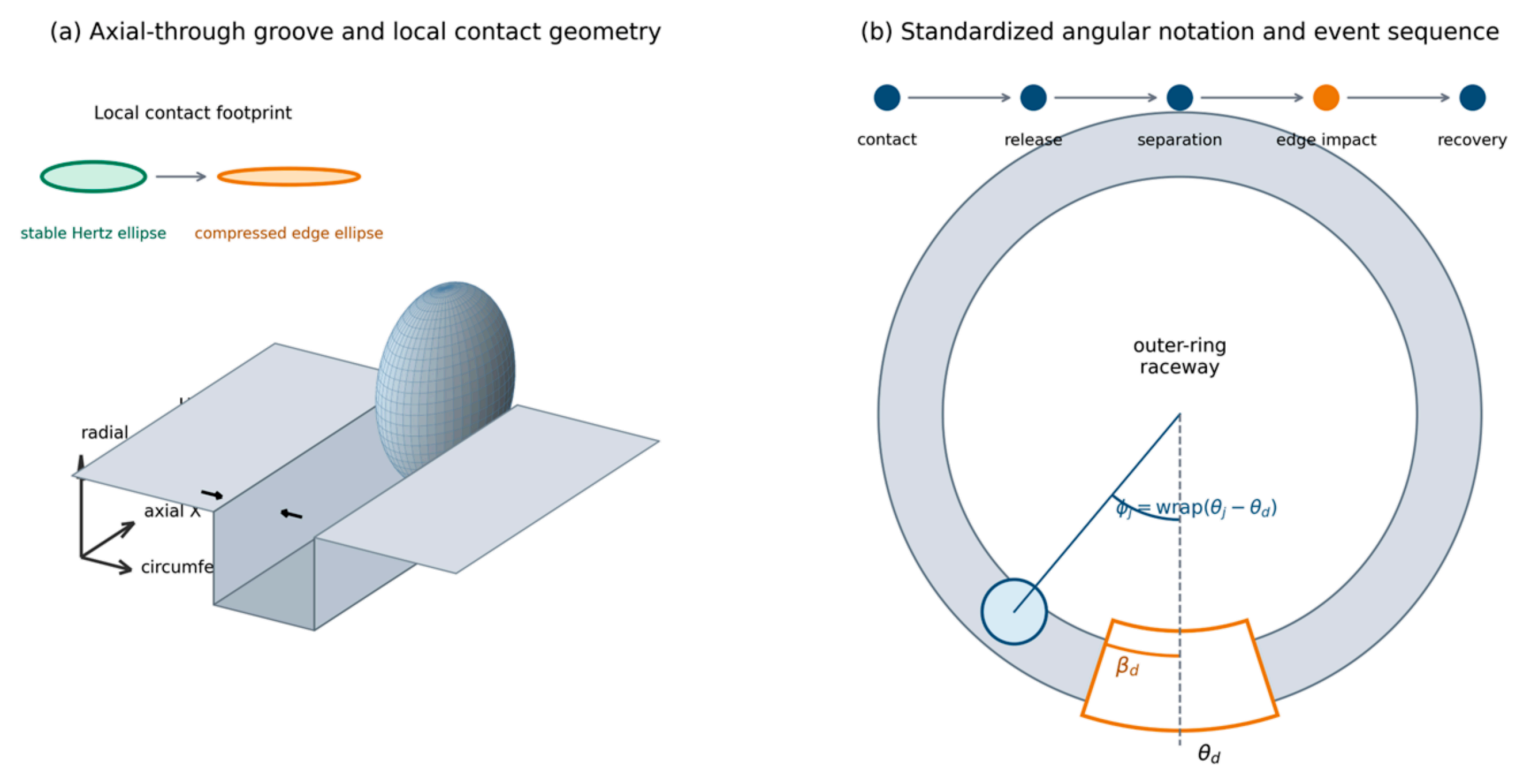
Three-dimensional axial-through groove geometry, standardized angular notation, contact-ellipse change, and event sequence during ball passage. The coordinate arrows in (**a**) indicate the axial, radial, and circumferential directions, while the arrows in (**b**) indicate the progression of the rolling element through the defect and the corresponding sequence of contact-state transitions.

**Figure 2:**
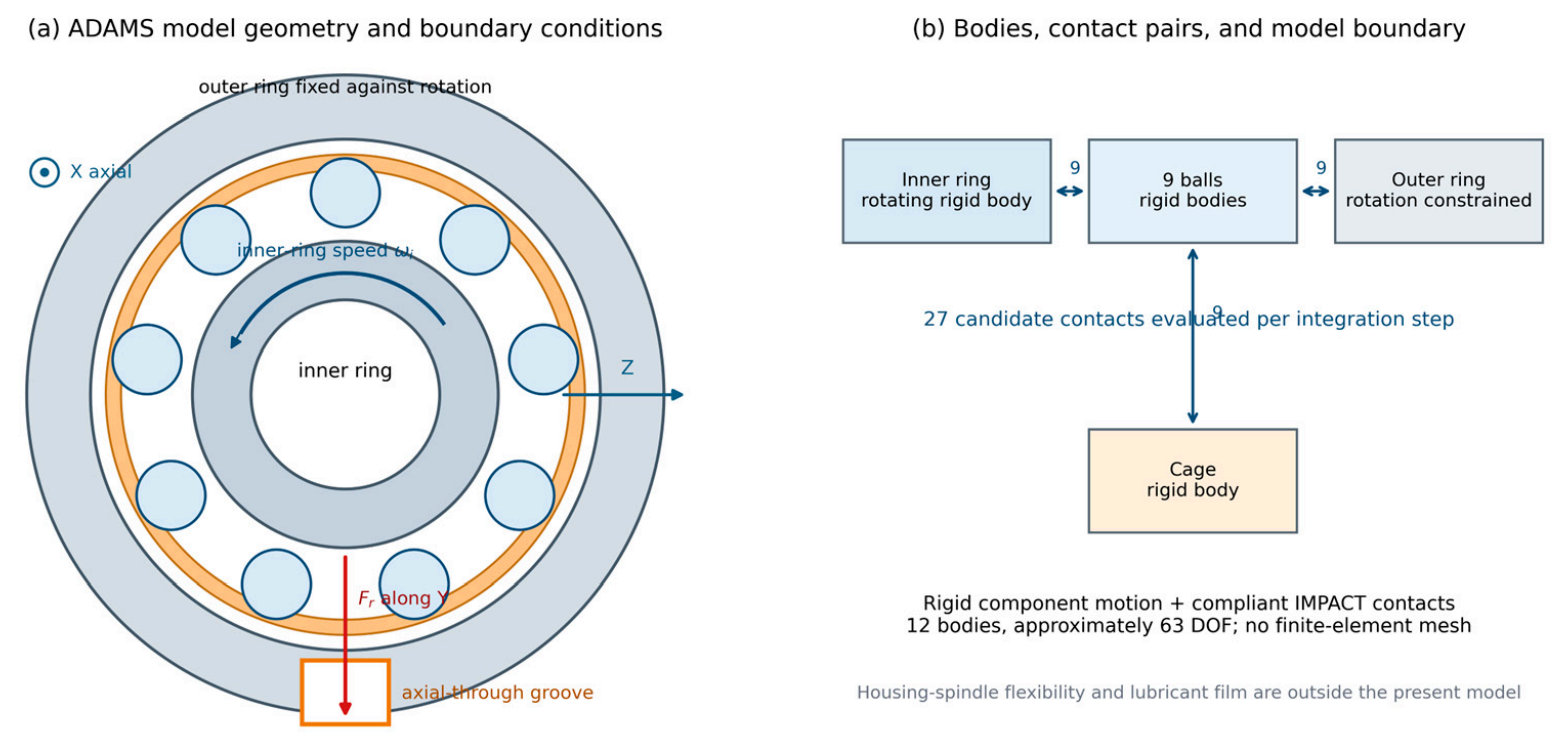
ADAMS model geometry, boundary conditions, component bodies, and compliant contact-pair topology for the SKF 6208 bearing.

**Figure 3:**
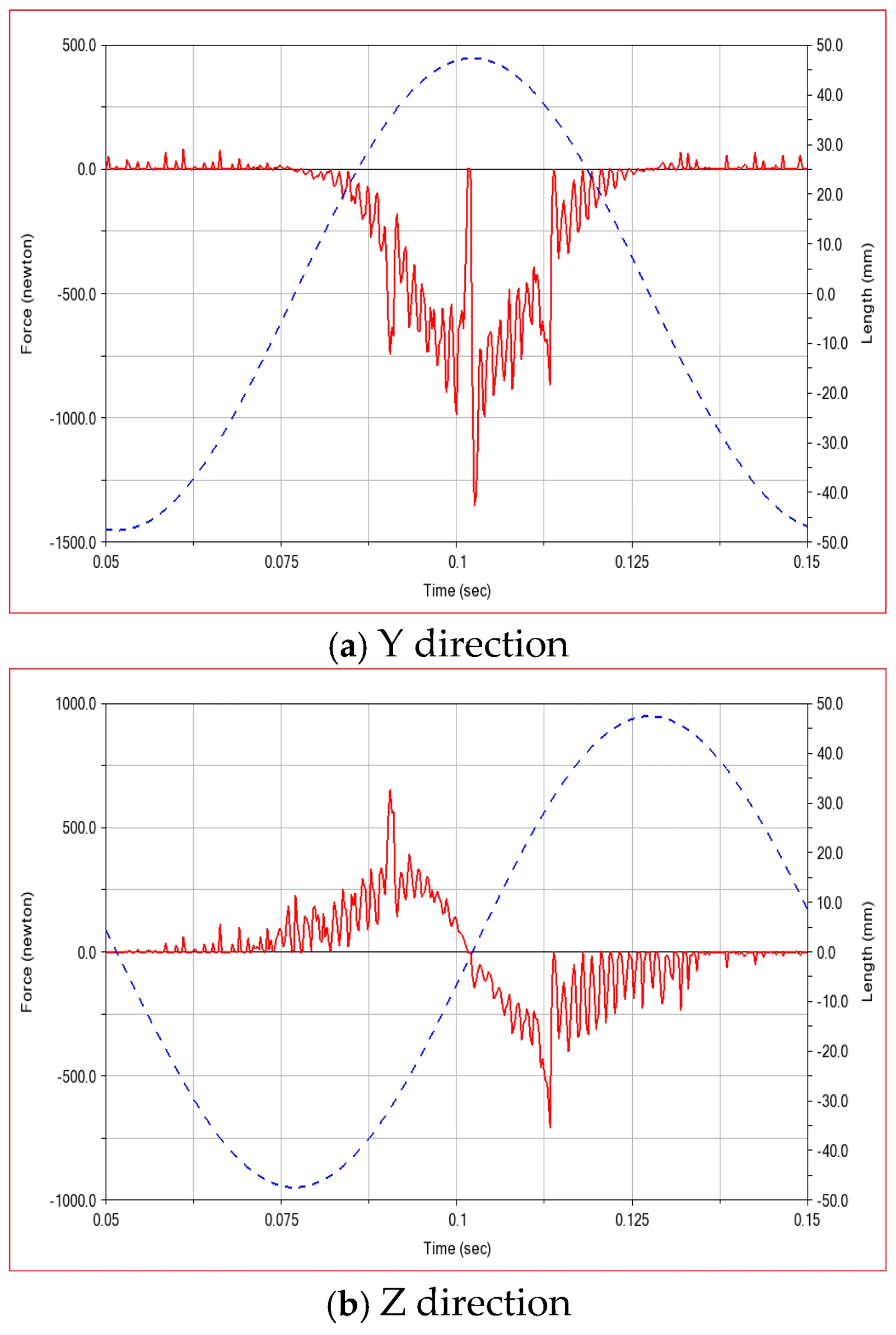
Contact force and ball trajectory during passage over the outer-ring raceway groove: (**a**) Y direction; (**b**) Z direction. The red solid line represents the contact-force component (left y-axis), and the blue dashed line represents the corresponding rolling-element trajectory component (right y-axis).

**Figure 4:**
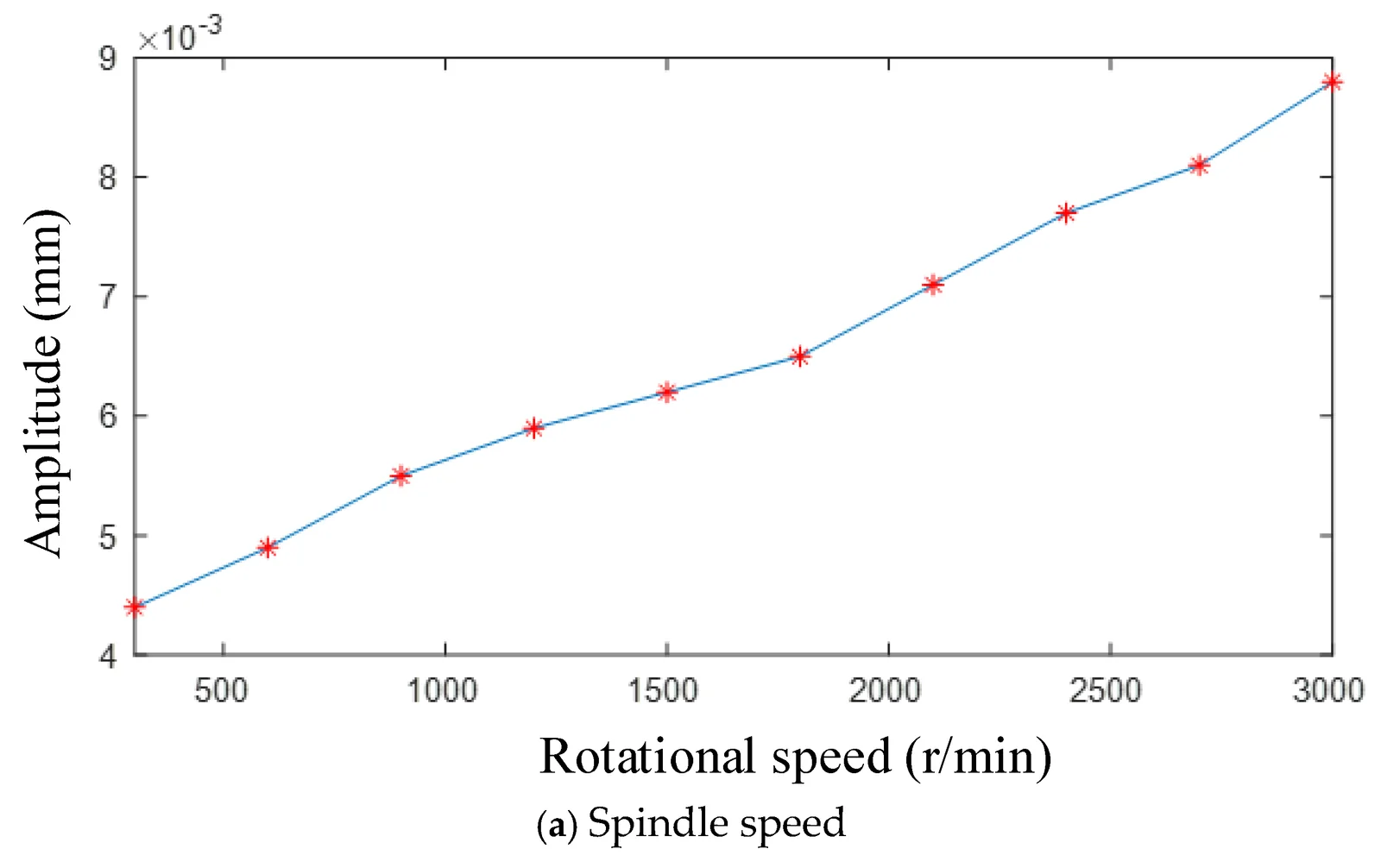

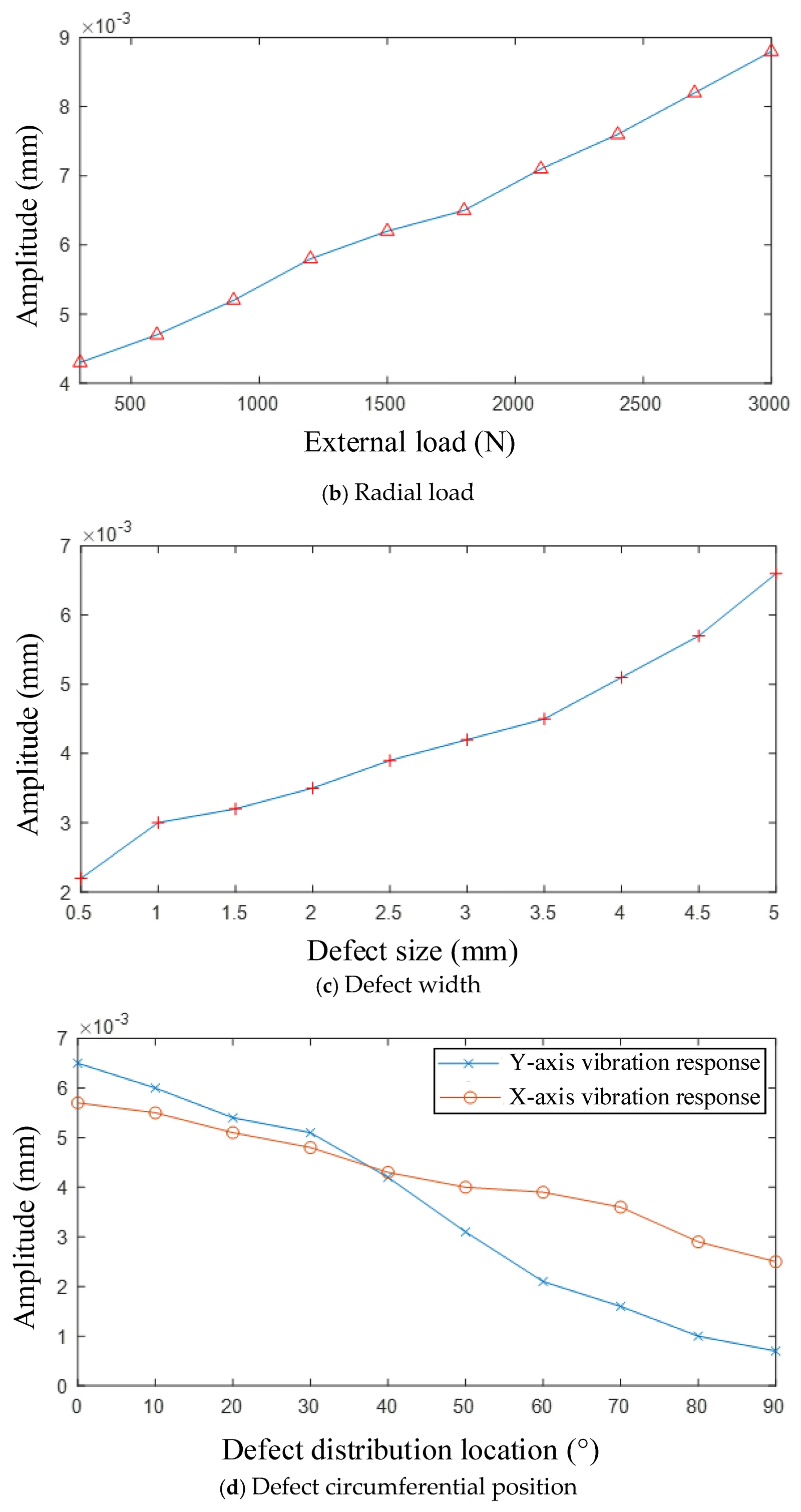
Effects of defect and operating parameters on defect-related spectral peak amplitude.

**Table 1:** Event states during passage over an outer-ring raceway groove.

Event Status	Judgment and Response Characteristics
Normal Contact	| φ_j | > β d, δ_j > 0 Rolling element and outer raceway stably transmit load
Entry/release	φ_j approaching the leading edge Contact stiffness decreases, load rapidly released
Short-term separation	*| φ_j | ≤ β d, δ_j ≤ 0* Outer raceway contact force close to zero
Trailing edge impact	*φ_j_* approaching the trailing edge with normal compression Causes transient impact and higher-order harmonics
Contact recovery	Passing beyond the trailing edge and *δ_j_* > 0 Load gradually restores to the normal cyclic state

**Table 2:** Main geometric parameters of the SKF 6208 bearing.

Parameter	Symbol	Value
Inner diameter/mm	*d*	40
Outer diameter/mm	*D*	80
Width/mm	*B*	18
Number of rolling elements	*N_b_*	9
Diameter of rolling elements/mm	*d_b_*	12
Pitch diameter/mm	*D_m_*	60

**Table 3:** Contact and friction parameters of bearing components.

Contact Object	*K*/(N·mm^−e^)	*e*	*C*/(N·s/mm)
Ball-inner/outer raceway	1.238 × 10^5^	1.5	123.8
Rolling element—cage	1.0 × 10^5^	2.2	10
Contact object	*d_max_*/mm	*μ_s_*	*μ_d_*
Ball-inner/outer raceway	0.01	0.10	0.06
Rolling element—cage	0.10	0.30	0.10

**Table 4:** Experimental pulse-period reconstruction for outer-ring raceway grooves at 1800 r/min.

Defect Width/mm	Theoretical Interval/ms	Reconstructed Mean Interval/ms	Pulse-Interval Relative Error/%
0.5	9.26	9.55	3.13
1.0	9.26	9.43	1.84
1.5	9.26	9.34	0.86

**Table 6:** Effects of defect and operating parameters on response characteristics.

Influencing Parameters	Characteristic Frequency	Spectral Peak Amplitude	Primary Mechanism of Action
Defect width increases	Basically unchanged	Monotonically increases	Loss of contact time prolongs; trailing edge impact intensifies
Circumferential offset increases	Basically unchanged	Overall decreases, dominant direction Y→Z	Local load-bearing magnitude and directional components change
Spindle speed increases	Increases linearly	Overall increases	Pass-through frequency and edge collision velocity increase
Radial load increases	Basically unchanged	Monotonically increases	Contact deformation and normal collision force increase

## Data Availability

The original contributions presented in this study are included in the article. Further inquiries can be directed to the corresponding author.
